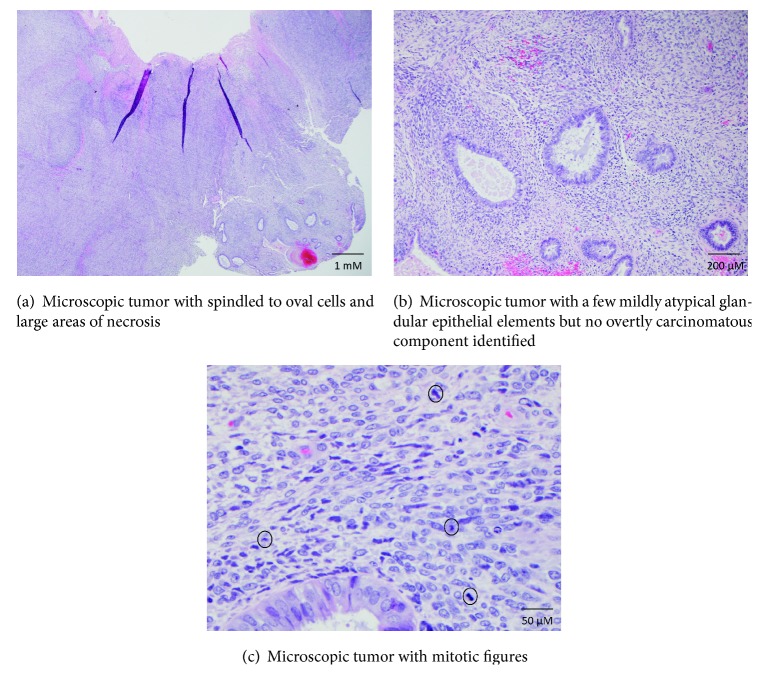# Uterine Sarcoma Presenting with Sepsis from* Clostridium perfringens* Endometritis in a Postmenopausal Woman

**DOI:** 10.1155/2018/8217296

**Published:** 2018-04-16

**Authors:** Mary J. Kao, Madhuchhanda Roy, Josephine Harter, Ryan J. Spencer

**Affiliations:** ^1^Department of Obstetrics and Gynecology, University of Wisconsin School of Medicine and Public Health, Madison, WI 53792, USA; ^2^Department of Pathology and Laboratory Medicine, University of Wisconsin School of Medicine and Public Health, Madison, WI 53792, USA; ^3^Division of Gynecologic Oncology, University of Wisconsin School of Medicine and Public Health, Madison, WI 53792, USA

## Abstract

*Clostridium perfringens* is an anaerobic gram positive rod that is found in normal vaginal and cervical flora in 1–10% of healthy women. Uterine infection with* Clostridium perfringens* is seen rarely but is often related to underlying uterine pathology and can progress quickly to sepsis. Early recognition of sepsis, prompt treatment with antibiotics, and source control with surgical management allow for optimal chance of recovery. We present a case of a postmenopausal woman who presented with sepsis, vaginal bleeding, and back pain who was found to have* Clostridium perfringens* infection in the setting of undifferentiated uterine sarcoma.

## 1. Introduction

Uterine sarcomas are stromal neoplasms that arise from dividing cells within the myometrium or from connective tissue elements within the endometrium. Uterine sarcoma accounts for 3 to 5% of all uterine malignancies [1]. Like other uterine malignancies, uterine sarcomas generally present with postmenopausal or abnormal uterine bleeding, abdominal pain, or pelvic pressure symptoms.

The prognosis for uterine sarcomas is generally poor compared to uterine carcinomas due to the aggressive nature of the tumor. The five-year survival rate ranges from 17 to 54% depending on the histopathological subtype [1]. We review a case of undifferentiated uterine sarcoma in a postmenopausal woman that initially presented as* Clostridium perfringens* sepsis, with the source of sepsis being endometritis in the setting of a large, necrotic tumor.

## 2. Case

This case involves a 79-year-old female G5P3023 who presented with* Clostridium perfringens* sepsis. She was in her usual state of health until two days prior to admission to an outside hospital with mental status changes and low back pain. She had fevers and her family reported that she seemed confused and was slow in response. She reported postmenopausal bleeding and lower abdominal pain that began a few days prior to admission. Her past medical history included history of deep venous thrombosis of her left lower extremity on lifelong anticoagulation, hypertension, hyperlipidemia, insulin resistance, hiatal hernia, and early stages of dementia. Her past surgical history was noncontributory. Her obstetrical history was significant for five pregnancies with three vaginal deliveries, a stillbirth, and a spontaneous abortion.

**Figure 1:**
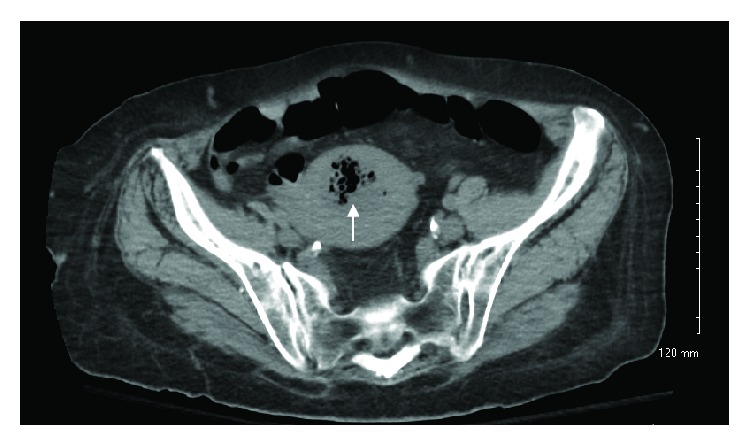
Axial CT image showing enlarged uterus with endometrial canal thickening and endometrial canal air (arrow) with surrounding inflammatory stranding, suggestive of endometritis.

On admission to the outside hospital, the patient was febrile to 103°F and white blood cell count (WBC) was 12,500 cells/*μ*L. A urinalysis was suggestive of a urinary tract infection so she was started empirically on vancomycin and ceftriaxone. Initial blood cultures grew gram positive bacilli, so metronidazole was added. CT of the abdomen and pelvis demonstrated an enlarged uterus with endometrial canal thickening and endometrial canal air with surrounding inflammatory stranding, suggestive of endometritis (Figure 1). Final blood cultures returned on HD3 as* Clostridium perfringens*, with the source presumed to be endometritis. Her antibiotics were changed to IV ertapenem. Follow-up blood cultures on HD2 and HD3 showed clearance of her bacteremia. She underwent dilation and curettage with an endometrial culture. Pathology revealed fragments of adenocarcinoma in a background of necrosis and the endometrial culture also grew* Clostridium perfringens, Bacteroides uniformis, *and scant* Peptoniphilus asaccharolyticus*. The patient was clinically stable and was transferred to our tertiary care center for management of her adenocarcinoma in the setting of* Clostridium perfringens* endometritis and septicemia after seven days at the outside hospital and seven days on IV antibiotics. Over time at the outside institution, her mental status improved and was clinically stable.

On evaluation after transfer, the patient felt well and denied pain or nausea. She was tolerating oral intake, voiding, having regular bowel movements, and ambulating. On exam, her vitals were normal and her BMI was 28.68 kg/m^2^. Her abdomen was soft, nondistended, and nontender with no rebound or guarding. Her extremities were warm and well perfused with significant venous stasis changes on bilateral lower extremities with associated skin breakdown. Her neurological exam was grossly intact. On pelvic exam, she had a smooth cervix and small 6-week sized mobile uterus, and no adnexal masses were appreciated. No purulent discharge was noted.

On admission to our institution (HD 7), laboratory values were significant for normal electrolytes, INR of 3.4, and white blood cell count of 10,000 cells/*μ*L. The IV ertapenem was continued. CT scan of the chest, abdomen, and pelvis was repeated, which showed a decrease in the gas component compared to the previous exam seven days earlier. The patient was clinically stable so preoperative planning was undertaken and included reversal of therapeutic anticoagulation. She received vitamin K on HD 7 with a repeat INR of 2.1. She was then given two units of fresh frozen plasma on HD 8 prior to surgery.

On HD 8, she underwent a total laparoscopic hysterectomy and bilateral salpingo-oophorectomy. Given her recent clinical condition, decision was made prior to surgery to forego lymphadenectomy which was discussed with patient. Intraoperative findings revealed purulence draining from the cervical os. The uterus was enlarged and globular, but mobile. Her fallopian tubes and ovaries were normal in appearance. There were no adnexal masses or evidence of metastatic disease. The procedure was uncomplicated and estimated blood loss was 300 cc. Intraoperatively, she received one unit of packed red blood cells due to acute blood loss superimposed on chronic anemia. Her preoperative hemoglobin was 8.2. After the procedure, she was transitioned from IV ertapenem to IV ceftriaxone and oral clindamycin per infectious disease consultant recommendations to provide excellent coverage for clostridial bacteremia and enteric gram negative rods in the immediate postoperative setting. Postoperative course was uncomplicated and she was discharged to a skilled nursing facility on HD 10/POD 2. She was discharged on oral ciprofloxacin with a plan for three additional days of treatment and oral clindamycin for fourteen additional days.

**Figure 2:**
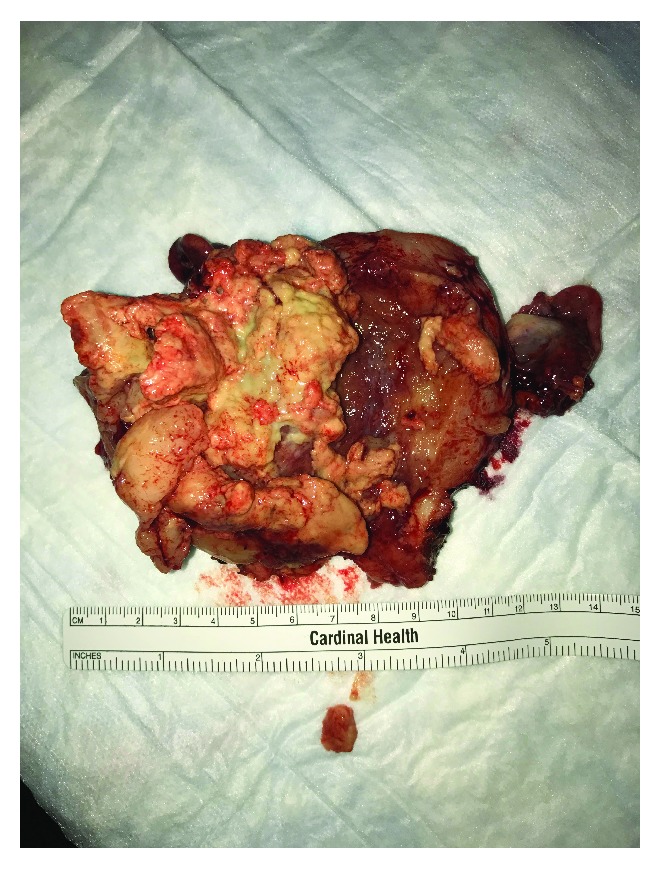
Gross specimen, bisected uterus and cervix, fallopian tubes, and ovaries, showing an exophytic, friable endometrial mass.

A picture of the bisected gross specimen is shown in Figure 2. The cervix is directly over the 8-9 cm marker of the ruler; the left tube and ovary are clearly visualized with the right tube visible at approximately 10 o'clock behind the necrotic intrauterine mass. Pathology revealed an undifferentiated uterine sarcoma mainly in the endometrium with invasion into the inner half of the myometrium. On gross examination, the endometrial cavity revealed an exophytic and friable mass (6.2 × 5.4 × 2.3 cm) located 2.2 cm from the lower uterine segment. Additional detached fragments of loose pink-tank, friable tumor mass (5.5 × 5.0 × 2.0 cm) were also submitted for histologic examination. The cervix, bilateral ovaries, and fallopian tubes were not grossly involved.

Microscopically, the tumor was centered in the endometrium and was composed entirely of spindled to oval cells (Figures 3(a)–3(c)). Mitoses were readily identified (Figure 3(c), circles). There were large areas of necrosis. Differential diagnostic considerations included carcinosarcoma (malignant mixed Müllerian tumor/MMMT), endometrial stromal sarcoma (both low and high grade variants), adenosarcoma with sarcomatous overgrowth, and leiomyosarcoma. While there were a few mildly atypical glandular epithelial elements, no overtly carcinomatous component was identified, militating against the diagnosis of carcinosarcoma (Figures 3(a) and 3(b)).

Immunohistochemically, the malignant cells demonstrated patchy positivity for cytokeratin AE1/AE3, ER (estrogen receptor), PR (progesterone receptor), CD10, cyclin D1, h-caldesmon, desmin, and SMA (smooth muscle actin) and were negative for myogenin, ALK, and DOG-1 (not shown). Fluorescence in situ hybridization (FISH) testing for* JAZF1*,* PHF1*, and* YWHAE* rearrangements was negative.

The pathology slides of the dilation and curettage from the outside hospital were reviewed and compared to the hysterectomy specimen. While specimens did contain some mildly atypical glandular epithelial elements, they were detached and did not have an overtly malignant morphology. When evaluated in the context of the hysterectomy specimen, which contained no carcinomatous component, we interpreted that glandular component as fragments of benign cervix/endometrium.

The patient was followed up in the office 5 weeks after surgery and had recovered well although still requiring intermittent nursing care at home. Adjuvant chemotherapy with gemcitabine and docetaxel was recommended at that time and arrangements were made to see a medical oncologist closer to her home. Ultimately, she was diagnosed with a second primary lung cancer a short time after her recovery which was not apparent on her imaging for this hospital admission. A few months later she died from what was thought to be diffuse metastatic disease with multiple lung masses by the physicians caring for her at that time.

## 3. Discussion

*Clostridium* species are anaerobic gram positive rods and are commonly found in nature in soil and marine sediments due to their ability to form endospores.* Clostridium* soft tissue infections are seen in wound contamination, anaerobic cellulitis, myonecrosis, and necrotizing fasciitis [2]. Clostridial myonecrosis, also known as clostridial gas gangrene, is a life-threatening muscle infection that often develops contiguously from an area of trauma or from hematogenous spread from the gastrointestinal tract with muscle seeding.

*Clostridium* species produce extracellular toxins known as alpha and theta toxins. Alpha toxins cause the absence of tissue inflammatory response by potently stimulating platelet aggregation and upregulating adherence molecules on polymorphonuclear leukocytes and endothelial cells. This causes a decline in muscle blood flow and ischemic necrosis due to the formation of occlusive intravascular aggregates composed of activated platelets, leukocytes, and fibrin. Theta toxin causes reduced systemic vascular resistance and increased cardiac output via induction of endogenous mediators such as prostacyclin and platelet activating factor that caused vasodilation [3, 4].

*Clostridium* myonecrosis progresses rapidly, often presenting with sudden onset severe pain at the site of infection with a mean incubation period of less than 24 hours. Signs of systemic infection include tachycardia, fever, shock, and multiorgan failure. Other complications of clostridial myonecrosis include jaundice, renal failure, hypotension, and liver necrosis. This was the case for our patient, who was in her usual state of health until two days prior to presentation when she started having lower back and abdominal pain. She had also been experiencing fevers in the 24 hours prior to admission and presented with altered mental status secondary to sepsis. Treatment includes early recognition of infection, surgical debridement, antibiotic therapy, and supportive measures [5]. Antibiotic therapy should include penicillin plus clindamycin or tetracycline. Our patient received antibiotics promptly and once the source of the infection was confirmed, prompt surgical removal of the infected organ was performed upon arrival to our institution. Prognosis is worse in patients who are already in shock at the time of diagnosis. In a series of 139 patients treated with antibiotics, surgery, and hyperbaric oxygen, 67 were in shock at admission, and all deaths (27 patients) occurred in this subset. Overall survival was 81% [6].

Clostridial myonecrosis is most commonly seen in wartime injuries or among victims of natural disasters due to delayed treatment of injuries. Less commonly,* Clostridium *infection is also seen in obstetrics, associated with abortion, retained placenta, intrauterine fetal demise, or prolonged rupture of membranes.* C. perfringens* is considered normal vaginal flora, found in the vagina and cervix in 1–10% of healthy women [7]. In the absence of infection,* C. perfringens* has no clinical significance and causes clinical illness in only about 5% of isolates [7].* C. perfringens *uterine infection, while rare, is most commonly associated with uterine instrumentation during procedures such as dilation and curettage or during the postpartum period—neither of which preceded this infection.

We were able to identify only six other cases of clostridial sepsis in postmenopausal women in the literature. These women also had underlying uterine pathology, including four cases with uterine endometrial adenocarcinoma [8–10] and two cases with degenerating uterine leiomyoma [11, 12]. Of the cases with endometrial adenocarcinoma, two were being treated with intrauterine radiocesium and the infection is thought to have been transmitted from one patient to the next. The third case was a spontaneous case of clostridial infection, who presented with sepsis similar to our patient but had progressed to the point of uterine perforation and was treated with emergency surgery.

In the current case, the underlying uterine sarcoma likely made the uterus more susceptible to infection, resulting in a quick progression to septicemia. The tumor had large areas of necrosis into the myometrium and vascular insufficiency, likely predisposing the site to infection. Clinically, sepsis was identified promptly and the patient was started on broad-spectrum antibiotics while the source of infection was investigated. Although surgical exploration and removal of the involved organs was ultimately undertaken, it was not until eight days after diagnosis. Ideally, surgery would have been undertaken immediately upon diagnosis or suspicion of a necrotizing soft tissue infection as source control is critical to patient outcome and optimizing antimicrobial therapy.

## Figures and Tables

**Figure 3:**